# Mentalizing in a movie for the assessment of social cognition (MASC): the validation in a taiwanese sample

**DOI:** 10.1186/s40359-023-01321-0

**Published:** 2023-09-22

**Authors:** Yu-Lien Huang, Tzu-Ting Chen, Isabel Dziobek, Huai-Hsuan Tseng

**Affiliations:** 1 Department of Psychology, Chung Shan Medical University, Taichung, Taiwan; 2 Department of Psychology, Fo Guang University, Yilan, Taiwan; 3 Department of Psychology, Humboldt-Universität zu Berlin, Berlin, Germany; 4 Institute of Behavioral Medicine, College of Medicine, National Cheng Kung University, Tainan, Taiwan; 5 Department of Psychiatry, College of Medicine, National Cheng Kung University Hospital, National Cheng Kung University, Tainan, Taiwan

**Keywords:** Emotion recognition, Mentalizing, Social cognition, Theory of mind

## Abstract

**Background:**

The present study evaluated the psychometrics properties of a sensitive video-based test used in the evaluation of mentalizing skills, that is, the Movie for the Assessment of Social Cognition-Taiwanese version (MASC-TW).

**Methods:**

We recruited two independent samples of nonclinical participants (N = 167) and adult patients with schizophrenia (N = 41). The MASC-TW and two other social cognition measures, namely the Chinese version of Theory of Mind task (ToM) and the Taiwanese version of the Diagnostic Analysis of Nonverbal Accuracy-2 (DANAV-TW-2), and an executive function measure of the Wisconsin Card Sorting Test (WCST), were administered to both groups.

**Results:**

The MASC proved to be a reliable measure of mentalizing capacity, high Cronbach’s α value of 0.87. The intraclass correlation coefficient for the MASC-TW total correct scores was 0.85 across three waves of data collection. Across the entire sample, the scores on the MASC-TW were significantly correlated with verbal and nonverbal scores for the ToM task and recognition of facial and prosodic emotion on the DANAV-TW-2. Both executive function and emotion recognition emerged as noteworthy predictors of mentalizing, indicating that these two variables might play crucial roles in the development of mentalizing capacities. Finally, a receiver operating characteristic analysis revealed that in patients with schizophrenia, the MASC was the most accurate discriminator of diagnostic groups, highlighting the validity of the MASC.

**Conclusions:**

Overall, the MASC-TW is an ecologically valid and useful tool for assessing mentalizing abilities in a Taiwanese population.

## Introduction

Social cognition refers to the ability to construct mental representations of oneself, others, and one’s relationships with others and to flexibly use those representations to guide social behaviors [1]. Social cognition comprises three key elements: (a) social perception, which involves perceptual processing of social cues, such as facial expressions; (b) social comprehension, which encompasses understanding others’ cognitive or affective states; and (c) social decision-making, which entails planning behaviors that consider both one’s personal and others’ goals [2]. The concept of mentalizing and its measurement in the social comprehension context have gained popularity in social cognition research over the past few decades [3, 4]. Mentalizing is “the cognitive process by which individuals implicitly and explicitly interpret their own actions and the actions of others as meaningful on the basis of intentional mental elements such as desires, needs, feelings, thoughts, beliefs, and fantasies” [5]. Difficulties in mentalizing commonly occur in various mental disorders, including conditions such as psychotic disorders [6] and personality disorders [7]. Impairments in mentalizing may contribute to the severity of symptoms. Therefore, to identify compromised mentalizing capabilities would enhance the understanding of the development and persistence of mental disorders.

Mentalizing capacity encompasses both cognitive and affective dimensions, including the ability to infer others’ cognitive states (cognitive mentalizing) and the ability to understand others’ emotional states through nonverbal cues, such as facial expressions (affective mentalizing) [5, 8]. Abu-Ake and Shamay-Tsoory [9] proposed that the cognitive and affective dimensions are served by separate interacting networks and their interaction within and between these two networks constitutes a broader mentalizing network. Therefore, both cognitive and affective dimensions should be included in the measurement of specific facets of the theory of mind (ToM) and mentalizing [10].

The Movie for the Assessment of Social Cognition (MASC) [11] was designed to enable assessment of social cognition through a video that displays social interactions between four characters, and it includes the following key components: a visual channel (i.e., facial emotion recognition and gaze), an auditory channel (i.e., prosodic emotion recognition), and a verbal channel (i.e., language content). Participants in studies involving the MASC are required to deduce the thoughts, emotions, and intentions of the characters in the video. Notably, the MASC has been demonstrated to effectively capture both the cognitive and the affective dimensions of social cognition [12]. Furthermore, scores derived from the MASC were reported to be significantly correlated with scores on the Reading the Mind in the Eyes Test in both nonclinical adolescents and nonclinical adults. This finding indicates that the MASC is a reliable measure of ToM and mentalizing capacities, particularly attributing emotional states to others [13]. The MASC is also a valid tool for differentiating between individuals without psychiatric disorders and those with neurodevelopmental or psychiatric conditions. Individuals with conditions such as schizophrenia or individuals with autism spectrum disorder (ASD) have been demonstrated to exhibit lower mentalizing abilities [14, 15].

According to the two-systems framework of ToM, understanding the mental states of others involves social-perceptual components (such as facial perception and recognition) and reflexive cognitive components (such as language and reasoning) [16, 17]. This framework highlights that both cognitive function and emotion recognition play pivotal roles in developing the ability to draw inferences about the mental states of others [18–20]. Empirical evidence obtained from a study revealed a positive association between neurocognition and social cognition in both healthy controls and patients with schizophrenia [21]. Moreover, a noteworthy direct effect of cognitive function and emotion recognition in predicting ToM has been observed [19, 20], highlighting the crucial roles of cognitive function and emotion recognition in the development of mentalizing capacities. Furthermore, a study noted a significant connection between executive function and emotion recognition and was able to use this connection to predict ToM capacity in patients with schizophrenia [20]. Building on this foundation, the present study investigated the contributing roles of executive function and emotion recognition in mentalizing and whether emotion recognition mediates the association between executive function and mentalizing.

### Purpose of the present study

The present study validated the applicability of the MASC–Taiwanese version (MASC-TW) for the Taiwanese population for the assessment of cognitive and affective metalizing capacities, which include the following: (1) cognitive mentalizing, which refers to the ability to infer others’ mental states, and (2) affective mentalizing, which refers to the ability to understand others’ emotional states. This study further proposed the following hypotheses: (1) the MASC-TW exhibits robust psychometric properties, including reliably and validity; (2) the MASC-TW can differentiate between individuals without psychiatric diagnoses and patients with schizophrenia; and (3) emotion recognition plays a mediating role in the association between executive function and mentalizing.

## Methods

### Participants

A total of 167 participants were recruited from an East Asian ethnic group in Taiwan (66.5% women, age range: 18 to 65 years [M = 31.34, SD = 14.13]). The sample comprised 126 healthy controls (69.8% women, age range: 18 to 65 years [M = 26.05, SD = 11.01]) recruited from the community through advertisements and 41 patients with schizophrenia (56.1% women, age range: 29 to 65 years [M = 47.61, SD = 9.49]) recruited from the day-care centers, community rehabilitation centers, and psychiatric outpatient clinics of a medical center in northern and southern Taiwan. The exclusion criteria for both groups were as follows: (1) a history of drug dependence or abuse, (2) a history of current or past general medical illnesses or any neurological conditions that may interfere with cognitive function, and (3) inadequate communication and language skills to comprehend the study instructions.

All patients included in the study fulfilled the diagnostic criterion for schizophrenia listed in the Diagnostic and Statistical Manual of Mental Disorder, Fifth Edition (DSM-V) [22]. The confirmation of a schizophrenia diagnosis was based on psychiatric records and a structured diagnostic interview, that is, the Mini-International Neuropsychiatric Interview (MINI) [23]. The inclusion criteria for patients with schizophrenia were as follows: (1) an age between 20 and 65 years, (2) stability of symptoms throughout the course of schizophrenia, and (3) no history of ongoing physical illness. The symptom severity was assessed using the Mandarin version of the Positive and Negative Syndrome Scale (PANSS-M) [24]. None of the participants were assessed during the acute phase of their condition (see Table 1).

**Table 1:** Demographic information and clinical characteristics of two groups

	Schizophrenia (*n* = 41) Mean ± SD	Healthy Control (*n* = 126) Mean ± SD	group comparison
Gender	18 males (56.1%) 23 females (43.9%)	38 males (30.2%) 88 females (69.8%)	*χ*^*2*^(1) = 2.62, *p* = .105
Age	47.61 (± 9.49)	26.05 (± 11.01)	*t(165) = 11.25, p < .001*
Positive symptoms	7.39 (± 2.10)	-	
Negative symptoms	12.12 (± 12.12)	-	
Disorganized symptoms	6.80 (± 2.24)	-	
Excited symptoms	5.76 (± 2.26)	-	
Depressed symptoms	5.22 (± 1.98)	-	
Executive function (WCST)	3.10 (± 3.20)	7.51 (± 2.27)	*t*(53.653) = -9.72, *p* < .001
MASC-TW correct	14.59 (± 4.83)	29.83 (± 4.15)	*t*(165) = -19.61, *p* < .001
Cognitive MASC-TW	8.24 (± 3.14)	18.02 (± 3.20)	*t*(165) = -17.08, *p* < .001
Affective MASC-TW	6.07 (± 2.40)	11.29 (± 1.91)	*t*(165) = -14.23, *p* < .001
MASC-TW exToM	6.63 (± 2.75)	6.34 (± 2.58)	*t*(165) = 0.62, *p* = .535
MASC-TW lessToM	15.63 (± 4.02)	5.54 (± 2.80)	*t*(53.211) = -14.95, *p* < .001
MASC-TW noToM	7.95 (± 3.10)	3.28 (± 2.08)	*t*(52.247) = -9.02, *p* < .001
ToM verbal score	20.66 (± 7.59)	26.83 (± 2.77)	*t*(43.508) = -5.10, *p* < .001
ToM nonverbal score	10.71 (± 3.57)	14.10 (± 3.16)	*t*(165) = -5.78, *p* < .001
DANVA-2-TW_Facial recognition	0.41 (± 0.18)	0.68 (± 0.11)	*t*(50.314) = -8.95, *p* < .001
DANVA-2-TW_Prosodic recognition	0.37 (± 0.20)	0.73 (± 0.13)	*t*(50.671) = -10.83, *p* < .001

*Note*. DANVA-2-TW = the Taiwanese Version of the Diagnostic Analysis of Non-verbal Accuracy 2; MASC-TW = the Taiwanese version of the Movie for the Assessment of Social Cognition; ToM = the Chinese version of the Theory of Mind task; WCST = the Wisconsin Card Sorting Test

No significant difference in sex distribution was noted between the two groups (*χ*^*2*^(1) = 2.62, *p* = .105). The patient group was significantly older than the control group [*t*(165) = 11.25, *p* < .001] and exhibited lower performance in executive function [*t*(53.653) = − 9.72, *p* < .001; Table 1].

### Procedure

After signed informed consent was obtained from the participants, the MASC-TW and the Chinese version of the ToM task [25] were administered to assess the participants’ mentalizing and ToM capabilities. The participants’ facial and prosodic emotion recognition abilities were assessed using a computerized facial and vocal expression subtest of the Taiwanese version of the Diagnostic Analysis of Nonverbal Accuracy 2 (DANVA-2-TW) [26]. Additionally, their executive function was assessed using the Wisconsin Card Sorting Test (WCST) [27]. Among the 126 normal controls, 55 completed a tendency of dispositional mindfulness assessment. Of the total participants, 89 underwent a second round of MASC-TW assessment, which was conducted 1–3 months (with an average interval of 52.61 days) after the first administration, and 18 underwent a third assessment, which was conducted 6 months after the first round. In recognition of their contributions to the study, each participant received compensation in the form of a base rate payment (US$5/hour) after each stage of assessment.

### Instruments

***MASC***. The English version of the MASC was developed by Dziobek et al. in 2006 [11]. The MASC is a sensitive video-based test designed to evaluate both cognitive and affective mentalizing capacities. The test involves a 15-minute video portraying two men and two women having dinner. This video depicts social situations involving misunderstanding, irony, body language, ambiguity, flirting, and insults. The MASC is presented to participants by using PowerPoint. At the start of the video, participants are told, “You are going to watch a 15-minute film, and you should attempt to understand what the characters are feeling and thinking.” Subsequently, the four characters are introduced using photographs and names. Participants are then instructed that the video depicts these characters gathering on a Saturday evening. As participants watch the video, it is paused 45 times. During each pause, participants are asked a multiple-choice question, which they must answer by inferring the thoughts, feelings, or intentions of the characters in the video. Samples questions include “Why do you think that Betty has made this comment?” and “How is Michael feeling?” To answer the questions, participants must draw upon information that has been presented in a verbal (understanding of verbal cues is required for 19 items, including 10 literal and 9 nonliteral items) or nonverbal (understanding of nonverbal cues is required for 16 items, including 6 items related to facial expression and 10 related to other nonverbal cues) format.

The MASC-TW is the Taiwanese, Mandarin equivalent of the original English MASC. A clinical psychology student translated the transcript of the English MASC into Chinese, and subsequently, another bilingual interpreter back-translated the Chinese version into English. A mental health expert then analyzed the translated Chinese version and the original English MASC to identify any discrepancies. The English MASC was retranslated iteratively until no discrepancy between the two versions could be identified.

The MASC presents participants with four response options for each question. One is correct (correct attribution of ToM to the characters of the film), and three are incorrect, with these incorrect answers involving excessive ToM (overmentalizing: attributing a mental state to a character even when the situation does not offer a mental explanation), reduced ToM (undermentalizing: misattributing a mental state), and no ToM (no-mentalizing: attributing a mental state to physical causation). All answers are scored using a standardized scoring key that reveals the correct and incorrect answers for each question. Overall success with respect to ToM performance is quantified using the total score for correct answers (maximum score = 45). MASC scores are also calculated using two subscales: cognitive ToM (26 items; maximum score = 26) and affective ToM (18 items; maximum score = 18) [12].

***DANVA-2-TW*** [28, 29]. The DANVA-2-TW is a validated, culturally tailored nonverbal assessment tool suited for the Han Chinese population in Taiwan. This instrument has demonstrated favorable interrater and test–retest reliability. To evaluate facial and prosodic emotion recognition abilities, the present study used 48 facial photos and 48 vocal clips conveying four basic emotions (12 items for the basic emotions of happiness, sadness, anger, and fearfulness) from the DANVA-2-TW. Each photo and clip had at least 80% agreement with the emotional categories. Each photo was displayed for 500 ms on a laptop screen in full-screen display with a resolution of 1024 × 768, and each vocal clip was played through earphones for a duration of 2–5 s. After being presented with each face conveying an emotion, the participants were asked to make a forced choice from the four emotion categories. The accuracy values were quantified as ratios of correct answers within each emotion category, with scores ranging from 0% (completely inaccurate) to 100% (completely accurate). To address potential response biases, this study adopted a correction method based on Wagner’s approach [30]. Wagner’s unbiased hit rate (Hu) is calculated as the product of the probability of detection and the frequency of hits. Hu is calculated as follows: Hu = (A_i_/B_i_) × (A_i_/C_i_), where A_i_ is the frequency of hits, B_i_ is the number of trials in which i is the target, and C_i_ is the frequency of i responses.

***Chinese version of the ToM task*** [25]. The Chinese version of the ToM task was used to assess the ability to understand other people by attributing mental states to both oneself and others. The original task incorporates elements such as false beliefs, faux pas, implication stories, and nonverbal tasks. In the current study, we focused on the faux pas and nonverbal tasks of the ToM task to measure verbal and nonverbal levels of ToM. The Chinese ToM task was demonstrated to have acceptable levels of reliability and validity in a Taiwanese control sample without a psychiatric diagnosis and a clinical sample of individuals with ASD.

***WCST*** [27]. In the present study, we employed a computerized version of the WCST to assess the ability to adapt to shifting patterns of reinforcement. The participants were required to match 48 response cards with four stimulus cards according to one of three dimensions (i.e., color, form, and number). To complete matches, the participants selected one of four number keys on a computer keyboard. The index of categories achieved was used for this study, with overall success being based on the number of times that 10 consecutive correct responses were made.

***MINI*** [23]. The MINI is a short, structured diagnostic interview designed to enable diagnosis of 16 axis I disorders outlined in the *DSM-IV* and 1 personality disorder. This instrument has satisfactory validity and reliability, with a diagnostic proficiency comparable to those of the Composite International Diagnostic Interview and the Structured Clinical Interview for DSM disorders.

***PANSS-M*** [24]. The PANSS-M is a 33-item semi-structured interview that is used to assess the presence and severity of positive and negative symptoms as well as general psychopathology among individuals with schizophrenia. Severity is rated on a scale with endpoints ranging from 1 (*absent*) to 7 (*extreme*). The PANSS-M has acceptable interrater reliability. We used the five-factor model proposed by Rodriguez-Jimenez and Bagney [31] to represent the following symptoms: positive symptoms (items P1, P3, P5, and G9), negative symptoms (items N1, N2, N3, N4, N6, and G7), disorganized symptoms (items P2, N5, and G11), excited symptoms (items P4, P7, G8, and G14), and depressive symptoms (items G2, G3, and G6).

***The Five Facet Mindfulness Questionnaire*** [32]. The Five Facet Mindfulness Questionnaire (FFMQ) is a 39-item self-report measure that is used to assess dispositional mindfulness on a 5-point scale with endpoints ranging from 1 (*never* or *very rarely true*) to 5 (*very often* or *always true*). Higher scores on the FFMQ indicate a greater tendency of dispositional mindfulness. The Chinese version of the TFFMQ [33], which was developed for a Taiwanese population, has demonstrated favorable reliability and validity.

### Statistical analysis

Data analyses were performed using SPSS, version 22.0. The demographic variables between groups were analyzed using independent *t* tests for continuous variables and chi-square tests for categorical variables. The internal consistency of the MASC-TW was assessed using Cronbach’s alpha and retest reliability. Pearson or Spearman correlations were used to assess the associations between MASC-TW scores and other study variables to determine convergent and discriminant validity. The sensitivity and specificity of the MASC-TW were evaluated using a receiver operating characteristic (ROC) curve analysis and by calculating the area under the curve (AUC). An AUC of 1.0 indicates that the diagnostic test is completely suitable for distinguishing between groups with and without disease [34]. The closer an AUC is to 1.0, the more accurate is the diagnostic test. Differences in mentalizing capacities between groups were analyzed using independent *t* tests. Finally, a path analysis of the association between executive function, metalizing capacities, and emotion recognition was conducted using Amos 16.0. Model fit was evaluated on the basis of several indicators: (1) a chi-square statistic (χ^2^), (2) the comparative fit index (CFI), and (3) the root mean square error of approximation (RMSEA) with a 90% confidence interval. For model fit evaluation, a nonsignificant chi-square statistic, CFI ≥ 0.95, and RMSEA ≤ 0.06 were considered to indicate an excellent model fit, and a nonsignificant chi-square statistic, CFI ≥ 0.90, and RMSEA ≤ 0.08 were considered to indicate an adequate model fit [35].

## Results

### Reliability

The internal consistency of the total correct scores on the MASC-TW were assessed using Cronbach’s alpha, with a value of 0.87 obtained for the entire sample, indicating the MASC-TW has satisfactory internal reliability. Among the 126 normal controls, 89 were invited to complete the MASC-TW a second time, with the results used to establish its test–retest reliability. The second round of the MASC-TW was completed 1–3 months after the first, with the average interval between the two rounds being 52.61 days. The mean correct scores for the first and second rounds were 29.84 (*SD* = 4.34) and 30.88 (*SD* = 4.63). The intraclass correlation coefficient (ICC) for the total correct scores for the MASC-TW was 0.85.

A total of 18 participants completed a third round of the MASC-TW, with the third round completed approximately 6 months after the first round (average interval between first and third round: 184.89 days). The mean correct scores for the first, second, and third rounds were 30.44 (*SD* = 3.40), 31.89 (*SD* = 4.07), and 32.17 (*SD* = 3.93). The ICC of the total correct scores on the MASC-TW was 0.85. This high ICC value indicates high agreement across different rounds for the whole sample.

### Validity

To further validate the MASC-TW, correlation analyses for the social cognition tests were conducted. In terms of construct validity, the results reveal significant positive associations. Correct MASC-TW scores exhibited significant positive correlations with both the verbal cue scores (*r* = .52, *p* < .001) and the nonverbal cue scores (*r* = .39, *p* < .001) of the ToM task. Additionally, the MASC-TW scores exhibited substantial positive correlations with facial (*r* = .68, *p* < .001) and prosodic (*r* = .71, *p* < .001) emotion recognition (with medium and large effect sizes; Table 2). Regarding discriminant validity, the correlation analyses revealed no significant correlation between correct MASC-TW scores and the sum scores of dispositional mindfulness (*r* = − .05, *p* = .702). Furthermore, no significant correlations were observed between the three types errors of the MASC-TW and total mindfulness scores (*r* = .01, *p* = .920; *r* = .05, *p* = .739; and *r* = − .15, *p* = .283 for no ToM, decreased ToM, and excessive ToM, respectively). The findings supported the validity of the MASC-TW as a robust measure of mentalizing capacity.

**Table 2:** Descriptive statistics and correlations among study variables (*N* = 167)

		1	2	3	4	5	6	7	8	9	10	11	12	
1	age	-												
2	Executive function (WCST)	− 0.44^***^	-											
3	MASC-TW correct	− 0.69^***^	0.62^***^	-										
4	Cognitive MASC-TW	− 0.68^***^	0.61^***^	0.96^***^	-									
5	Affective MASC-TW	− 0.57^***^	0.52^***^	0.89^***^	0.73^***^	-								
6	MASC-TW exToM	0.03	− 0.17	− 0.24	− 0.27	− 0.16	-							
7	MASC-TW lessToM	0.66^***^	− 0.55^***^	− 0.90^***^	− 0.87^***^	− 0.78^***^	− 0.07	-						
8	MASC-TW noToM	0.55^***^	− 0.46^***^	− 0.76^***^	− 0.69^***^	− 0.74^***^	− 0.12	0.58^***^	-					
9	ToM_verbal	− 0.55^***^	0.52^***^	0.52^***^	0.49^***^	0.48^***^	− 0.13	− 0.42^***^	− 0.48^***^	-				
10	ToM_nonverbal	− 0.39^***^	0.45^***^	0.39^***^	0.37^***^	0.36^***^	− 0.02	− 0.37^***^	− 0.32^***^	0.46^***^	-			
11	DANVA-2-TW_Facial recognition	− 0.57^***^	0.58^***^	0.68^***^	0.63^***^	0.66^***^	− 0.19	− 0.59^***^	− 0.51^***^	0.63^***^	0.36^***^	-		
12	DANVA-2-TW_Prosodic recognition	− 0.72^***^	0.63^***^	0.71^***^	0.66^***^	0.68^***^	− 0.12	− 0.66^***^	− 0.53^***^	0.66^***^	0.44^***^	0.74^***^	-	
Mean		31.34	6.43	26.09	15.62	10.01	6.41	8.02	4.433	25.32	13.27	0.61	0.65	
SD		14.13	3.16	7.87	5.28	3.04	2.61	5.36	3.11	5.17	3.57	0.18	0.22	

*Note*. DANVA-2-TW = the Taiwanese Version of the Diagnostic Analysis of Non-verbal Accuracy 2; MASC-TW = the Taiwanese version of the Movie for the Assessment of Social Cognition; ToM = the Chinese version of the Theory of Mind task; WCST = the Wisconsin Card Sorting Test

* Adjusted *p* values of 0.001 for multiple correlations

### Discrimination between the schizophrenia group and control group

The following AUC values were obtained: 0.987 for the MASC-TW, 0.788 for the ToM verbal score, 0.768 for the ToM nonverbal score, 0.884 for the DANVA-2-TW facial emotion recognition score, and 0.939 for the DANVA-2-TW prosodic emotion recognition score. Higher AUC values indicated better performance on the test. Compared with the other two social cognition measures, the MASC-TW had an AUC closer to 1.0, which indicates it had nearly perfect diagnostic performance in differentiating between patients with schizophrenia and normal controls. The DANVA-2-TW had the next highest AUC, followed by the ToM task.

In terms of group differences in mentalizing capacities, compared with the control group, the patient group had lower scores, including lower cognitive MASC-TW scores [*t*(165) = − 19.61, *p* < .001], affective MASC-TW scores [*t*(165) = − 19.61, *p* < .001], ToM verbal scores [*t*(43.508) = − 5.10, *p* < .001], ToM nonverbal scores [*t*(165) = − 5.78, *p* < .001], accuracy of facial emotion recognition [*t*(50.314) = − 8.95, *p* < .001], and accuracy of prosodic emotion recognition [*t*(50.671) = − 10.83, *p* < .001]. Furthermore, the patient group exhibited greater difficulties in mentalizing capacities, as evidenced by this group having more errors in the MASC-TW task, including errors related to decreased ToM [*t*(53.211) = − 14.95, *p* < .001] and no ToM [*t*(52.247) = − 9.02, *p* < .001; Table 1].

### Associations between executive function, emotion recognition, and mentalizing

**Fig. 1:**
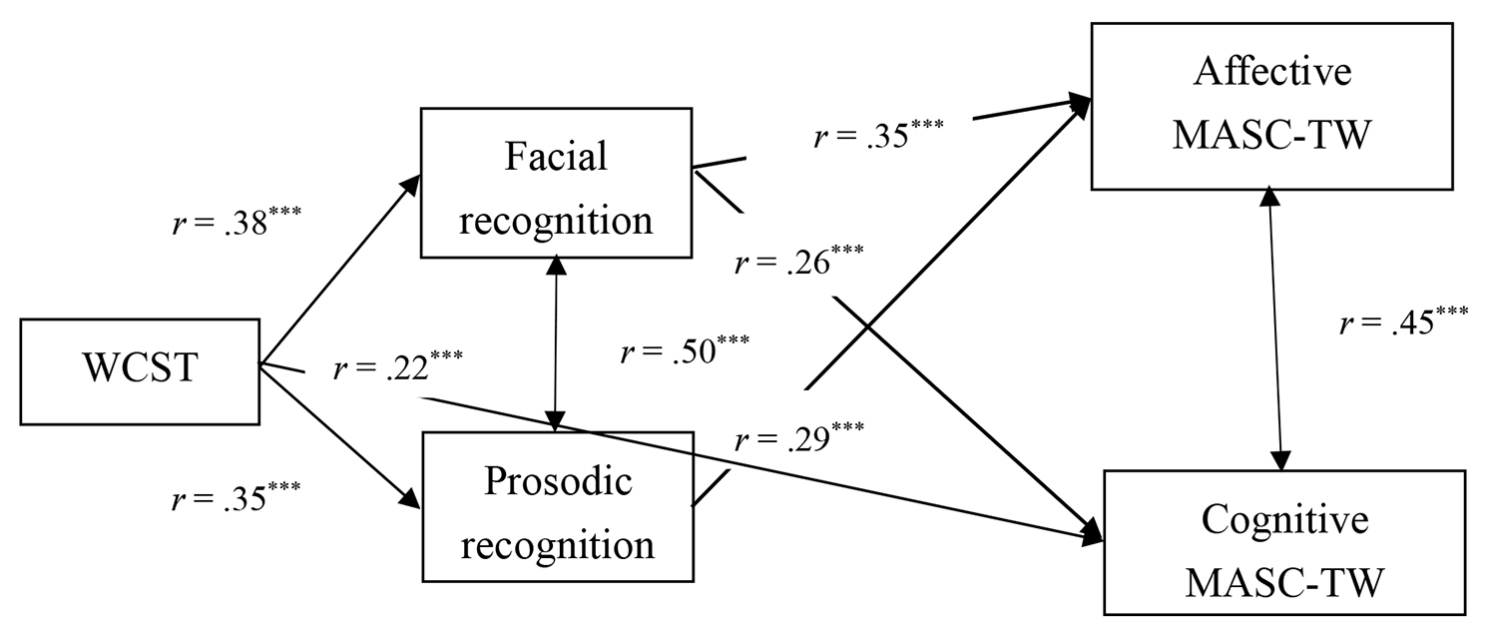
The path from neurocognition to mentalizing capacity via emotion recognition after controlling age. (Note: MASC-TW = the Taiwanese version of the Movie for the Assessment of Social Cognition; WCST = the Wisconsin Card Sorting Test. ^**^p < .01; ^***^p < .001)

The correlation analyses revealed a significant positive correlation between the correct MASC-TW scores and WCST scores (*r* = .62, *p* < .001). To further investigate this correlation, this study tested a hypothesized model by using Amos 16.0. The model included a pathway from executive function (WCST scores) to mentalizing capacity through emotion recognition. The model also controlled for age. The results of the model testing revealed an adequate model fit to the data [χ^2^ (1, *N* = 167) = 1.66, *p* = .435, CFI = 1.000, RMSEA = 0.000, 90% CI (0.000, 0.146); Fig. 1]. The hypothesized model revealed three significant direct paths: WCST scores had an effect on facial emotion recognition, WCST scores had an effect on prosodic emotion recognition, and WCST scores had an effect on cognitive MASC-TW scores. Additionally, the model revealed two indirect paths: WCST scores had an effect on cognitive MASC-TW scores through facial emotion recognition, and WCST scores had an effect on affective MASC-TW scores through facial and prosodic emotion recognition (Fig. 1). A bias-corrected bootstrapping procedure with 95% confidence intervals was employed to ascertain the significance of these indirect effects. The results reveal that all the outlined indirect effects were statistically significant (Table 3). This finding indicates that both executive function and emotion recognition significantly predict cognitive and affective mentalizing and that emotion recognition mediates the association between executive function and cognitive and affective mentalizing.

**Table 3:** Standardized estimates and 95% confidence intervals (CI) of each path in path analysis

Path	β	95% CI	*R* ^*2*^	
WCST to Facial recognition	0.38^**^	0.218, 0.569	0.43	
WCST to Prosodic recognition	0.35^**^	0.206, 0.498	0.60	
WCST to Cognitive MASC-TW	0.22^*^	0.100, 0.344	0.58	
WCST to Cognitive MASC-TW via Facial recognition	0.10^**^	0.068, 0.387		
Facial recognition to Cognitive MASC-TW	0.26^**^	0.113, 0.402	0.52	
WCST to Affective MASC-TW via Facial and Prosodic recognition	0.24^**^	0.149, 0.376		
Facial recognition to Affective MASC-TW	0.35^**^	0.191, 0.547		
Prosodic recognition to Affective MASC-TW	0.29^**^	0.146, 0.531		

*Note.* MASC-TW = the Taiwanese version of the Movie for the Assessment of Social Cognition; WCST = the Wisconsin Card Sorting Test

^*^p < .05; ^**^p < .01

## Discussion

The MASC was demonstrated to be a reliable measure of mentalizing capacity, with a Cronbach’s α value of 0.87. Moreover, it demonstrated favorable time stability, with an ICC of 0.85 obtained across three waves of administration. For the entire sample, the MASC scores exhibited significant correlations with the ability to understand ToM (as assessed using the ToM task) and nonverbal emotion recognition (DANAV-2-TW). Moreover, this study revealed both executive function (measured using WCST) and emotion recognition to have predictive roles in cognitive and affective mentalizing. Additionally, in patients with schizophrenia, the MASC-TW demonstrated high discriminative accuracy in distinguishing between diagnostic groups, which was confirmed using a ROC analysis. Taken together, these results reveal that the MASC-TW is an ecologically valid and useful tool for assessing mentalizing in a Taiwanese population.

The MASC-TW was demonstrated to be a reliable measure of the cognitive and affective dimensions of mentalizing. Notably, a significant correlation was identified between these cognitive and affective dimensions (*r* = .73), and both dimensions were significantly correlated with the verbal and nonverbal scores of the ToM task and facial and prosodic emotion recognition of the DANAV-TW-2. Moreover, the study findings reveal that executive function predicted affective mentalizing through the mediating effect of facial and prosodic emotion recognition and that executive function predicted cognitive mentalizing solely through the mediating effect of facial emotion recognition. These findings offer support for previous hypotheses indicating cognitive and affective mentalizing involve overlapping neural networks [9, 36] but also reveal that each dimension involves distinct neural pathways. The combination of these two dimensions forms the larger mentalizing network.

In the current study, prosodic emotion recognition did not predict cognitive mentalizing. Alba-Ferrara et al. [37] reported that the medial prefrontal cortex is activated in response to complex emotional tasks that involve inferring others’ social intentions and mental states but not in response to tasks that involve inferring simple emotions, such as fear, happiness, and anger. This is because although drawing inferences about mental states can be used to comprehend simple emotions, such inferences are not necessary to do so. This might explain why prosodic emotion recognition does not predict cognitive mentalizing.

In the current study, both executive function and emotion recognition contributed to mentalizing, with a pathway identified from executive function to both cognitive and affective mentalizing through emotion recognition. According to the two-system framework of ToM [19], understanding the mental states of others is a complex process involving both social-perceptual and reflexive cognitive components [16, 17]. That is, both explicit-level cognitive function and implicit-level emotion recognition enhance an individual’s ability to make inferences about the mental states of others [18–20], which indicates cognitive function and emotion recognition play vital roles in the development of mentalizing abilities.

The current study findings have notable clinical implications. Studies have consistently identified social cognition to play a pivotal role in determining functional outcomes [38], particularly in domains related to emotion perception and ToM [39]. For example, the potential of social cognition training programs in improving functional outcomes, such as social cognition skills, has been demonstrated in patients with schizophrenia [1] and ASD [40]. Thus, interventions that target the core domains of social cognition, including emotion processing, ToM, and mentalizing, have the potential to improve functional outcomes in patients with mental disorders.

The present study has several limitations that warrant consideration. First, the study had a cross-sectional design. Therefore, potential causal associations among the study variables could not be identified. Consequently, the study findings should be generalized with caution, and further research conducted using a longitudinal study design is required. Second, clinical information regarding the patients with schizophrenia, including information regarding their medication use and illness onset and duration, were not collected in this study, and therefore, the confounding effects of these variables could not be investigated.

## Conclusions

The current study offers valuable insights into the effectiveness of using the MASC-TW for assessing the cognitive and affective dimensions of mentalizing in a Taiwanese population. Furthermore, this study reveals the overlapping and distinct associations between cognitive and affective mentalizing, and the study findings indicate that the interaction between the two dimensions forms a mentalizing network.

## Data Availability

All data of this study are not publicly available to maintain the privacy of the individuals’ identities. The dataset is available from the corresponding author on reasonable request.
